# The Implicit and Explicit Prosody of the Focus Operator *yinwei* (Because) in Chinese Complex Sentences

**DOI:** 10.3390/bs15060766

**Published:** 2025-06-02

**Authors:** Liang Zhang

**Affiliations:** School of Foreign Studies, China University of Political Science and Law, Beijing 100088, China; cu008544@cupl.edu.cn

**Keywords:** Implicit Prosody Hypothesis, Chinese causal complex, focus operator *yinwei*

## Abstract

This study investigated the prosodic realization of the focus effect induced by the conjunction *yinwei* (because) in Chinese causal complex sentences. Although previous research has demonstrated the focal effect of *yinwei*, its prosodic manifestations remain unclear. Guided by the Implicit Prosody Hypothesis, in this study, we explored both implicit and explicit prosodic patterns across different conjunctions and linear-order conditions. Thirty Mandarin Chinese speakers (mean age = 20.2, gender-balanced) participated in silent and spoken reading tasks. Statistical analyses, including chi-square tests and Linear Mixed-Effects Models, were employed to assess the focus assignment and prosodic realization. The results indicate a consistent and robust focus effect of *yinwei*, marked by the primary stress on the succeeding cause clause in both implicit and explicit prosody. These findings enhance our understanding of the interplay between syntax and prosody in Chinese causal complex sentences.

## 1. Introduction

Causality underpins human cognition and discourse: readers and listeners constantly infer why events occur and what follows, integrating linguistic cues with world knowledge. While causal connectives have been extensively studied in languages with overt grammatical markers (Millis & Just, 1994; T. Sanders, 2005; T. J. M. Sanders & Noordman, 2000), their use in paratactic languages such as Mandarin Chinese warrants further investigation. In Chinese, the rare use of the conjunction *yinwei* (because) and the clause-order variations (cause–effect and effect–cause) serve as critical mechanisms for encoding causality, as well as the speakers’ pragmatic intention (L. Zhang, 2022). Despite advances in psycholinguistic research on Chinese causal devices, the prosodic implications of *yinwei* in complex sentences remain insufficiently addressed. This study aims to fill this gap by exploring how *yinwei* modulates focus, guided by the Implicit Prosody Hypothesis (IPH, Fodor, 1998, 2002).

In this study, three tightly interlinked goals were pursued. First, we sought to identify the acoustic realization of pragmatic focus on the *yinwei*-led cause clause in controlled readings by native Chinese speakers. Second, we aimed to uncover whether readers projected an analogous prosodic focus during the silent reading of *yinwei* sentences, using a judgement task to detect the implicit prosody. Third, we compared explicit (acoustic) and implicit (reading) prosodic patterns to evaluate the scope of the IPH within complex causal constructions.

This research fills a critical gap in the literature by integrating the study of conjunctions, clause order, and prosody in Chinese causal complex sentences, an area underexplored in tonal and paratactic languages. Moreover, it tests the universality of the IPH in a non-Indo-European context, contributing to broader theories of language processing. The findings also have practical implications for teaching Chinese as a second language, where prosodic cues are essential for discourse coherence. By combining acoustic and behavioral data, this study offers a novel, holistic approach to understanding prosody’s function in language. The results may further deepen our understanding of paratactic language processing in NLPand inform applications in language pedagogy.

## 2. Literature Review

Causality is a fundamental cognitive function, enabling humans to identify and attribute event causes (T. Sanders, 2005). Causality, in its broadest sense, is claimed to play “a pivotal role in explaining cognitive complexity” (T. Sanders, 2005), and language users seem to “have a preference for connecting information causally” (Noordman & Vonk, 1998). The inference of causality between events and statements has also been found to be significant in reading, especially in oral communication. Although many of the causal relations in discourse are implied and must be inferred by the reader, in languages, there are multiple strategies for expressing causality, for example, the conjunction and linear order of complex causal sentences. We begin by exploring how causality is expressed in discourse, focusing on the roles of conjunctions and linear order across languages.

### 2.1. Causality in Discourse

In many languages, connectives such as “because”, “therefore”, or “so” explicitly signal logical relationships, aiding in the construction of mental models (Zwaan & Radvansky, 1998). Conjunctions are assumed to provide processing instructions that specify the enfolding discourse structure and further infer logical relations between words, sentences, and even paragraphs (Gernsbacher, 1997; L. Zhang et al., 2018; L. Zhang, 2022). As significant markers of semantic functions, conjunctions play a major role in maintaining discourse coherence and thus in text processing and understanding (T. J. M. Sanders & Noordman, 2000). Such markers are proven to have a positive impact in discourse processing on recall tasks (Lorch & Lorch, 1986), summarization tasks (Hyönä & Lorch, 2004), and categorization tasks (McNamara et al., 1996; T. Sanders et al., 2007). These studies also observed improved performance in terms of the precision of recalled details (Loman & Mayer, 1983), the accuracy of responses to text-related queries (Degand & Sanders, 2002; Millis & Just, 1994), and the celerity of responses during verification tasks (T. Sanders et al., 2007). In real-time processing, causal connectives facilitate readers’ real-time processing of sentences expressing relationships between events (Canestrelli et al., 2016; Millis & Just, 1994; Silfhout et al., 2014, 2015).

While causal connectives are common in many languages, Mandarin Chinese employs a distinct paratactic structure that shapes its discourse differently. In Mandarin Chinese, the expression of causality operates differently due to its nature as a paratactic language. Unlike Indo-European languages, which are typically hypotactic and rely on conjunctions to illustrate specific relations within sentences, Mandarin Chinese often omits such connectives. Instead, it juxtaposes clauses side by side, leaving relationships such as causality to be inferred from the context, word order, or semantic cues (N. Li & Thompson, 1981). For example, a sentence might present a cause followed by its effect without any conjunction, relying on the semantics alone to imply the connection.

This paratactic structure marks a stark contrast to Indo-European languages. In English or French, conjunctions explicitly clarify causal links—e.g., “She was tired because she worked late”. In Chinese, a similar idea might simply be *ta1 gong1 zuo4 dao4 hen4 wan3*, *lei4 le5* (She worked late, tired), with the causal relationship understood implicitly. This difference influences how causality is conveyed and processed in Chinese discourse. Without overt markers, listeners or readers depend more heavily on contextual understanding to interpret the intended meaning.

The implications of this linguistic trait are significant. The preferred order of Chinese causal sentences has long been the subject of considerable debate. Although traditional grammar describes that the cause–effect sequence is preferred in Mandarin complex sentences (Hou, 1998; X. Q. Li, 2003; Xing, 2001; X. Zhang, 2015), as it aligns with the characteristic placement of adverbial clauses (Xing, 2001; Chao, 1979) and illustrates a broad iconic relationship with the actual sequence of events (Dai, 1991), empirical and corpus-based studies (L. Zhang et al., 2018) have yielded mixed results, some supporting the predominance of the cause–effect order in both spoken and written language (Biq, 1995; Song & Tao, 2008), while others indicate that the effect–cause order has an advantage in spoken language (H. Li, 2012; Wang, 2002; Xiao, 2010).

In summary, the paratactic nature of Mandarin Chinese sets it apart from Indo-European languages and shapes its discourse in unique ways. This characteristic enriches our understanding of causality, showing how linguistic structure influences both expression and interpretation. Building on this foundation, we now examine how the rare use of causal conjunctions as a focus operator enhances the salience of causal clauses, particularly in Chinese complex sentences.

### 2.2. Focus in Complex Sentences

The relationship between the narrative order and the focus of complex sentences remains unclear. By the focus of complex sentence, we mean the clause as a broad focus that is more important semantically and pragmatically. The general view of the focus of Chinese complex sentences is that the focus of a pivotal complex sentence lies in the main clause (Huang & Liao, 2007), i.e., the effect clause in causal complex sentences. For example, in *yinwei ta1 e4 le5*, *ta1 chi1 le5* (because he was hungry, he ate), speakers present the causal complex in a cause–effect order, with the effect clause containing the pivotal information. This follows the natural temporal sequence of the cause leading to the result and positions the result as the discourse’s informational highlight.

By contrast, Li (2018) argues that the cause clause often becomes the true focal point. Taking the same sentence as an example, the cause clause (*ta1 e4 le5*, he was hungry) is the focus clause. This is because the result is often apparent, whereas the cause leading to the result is often unclear, necessitating an exploration of the cause based on the result.

Some scholars also argue that the focal position in causality sentences is not fixed; different clause orders reflect different focal positions and vary in spoken and written language (Chen, 1984; Lu, 1980; Lv, 1994; H. X. Mo & Zhang, 2001; Zhu, 2013). In a recent corpus analysis of Chinese spoken discourse, the conjunction is preferably not used to express causal relations (91%) (L. Zhang, 2022). At the same time, the exclusive use of the conjunction *yinwei* (because) is only observed to be placed at the end of the narrative sequence to highlight the content of the cause clause, thereby making the cause clause the focal point of the discourse. That is, the speaker’s use of the causative conjunction *yinwei* (because) emphasizes the expression of the discourse focus to a certain extent. Cross-language evidence also supports the pragmatic focus effect (Ford, 1993, 1994; Ford & Mori, 1994). This further motivates us to investigate its prosodic realization.

### 2.3. Prosody as a Pragmatic Cue

One of the most prominent methods used to signal pragmatic meaning in spoken discourse is prosody. Prosody, which refers to acoustic variations in speech, carries various meanings and functions in daily communication (Cole, 2015; Cutler et al., 1997; Hirschberg et al., 2020; Wagner & Watson, 2010). Prosody marks discourse and rhetorical structure (Hirschberg & Grosz, 1992; den Ouden et al., 2009; Swerts, 1997; Tyler, 2013; L. Zhang et al., 2014, 2016), marks focus and information status (Brown, 1983; Büring, 2006; Su & Tseng, 2018; Wen et al., 2018), and conveys attitudinal meanings (Mitchell & Ross, 2013). Furthermore, research has shown that prosodic information contributes to speech processing. Many studies have found that prosodic information is essential for spoken language production and perception (Jusczyk et al., 1999; Kentner, 2012; Morgan, 1996; Snedeker & Trueswell, 2003; Tyler, 2013). Speakers use various prosodic patterns to convey pragmatic values that add up to the core semantic meanings of spoken units or segments. This is also a clue provided by the speaker to decode communicative intentions (House, 2006). The pragmatic focus formed by the conjunction *yinwei* allows us to conduct an acoustic experiment to examine the explicit prosody of Chinese causal complex sentences with the presence or absence of the conjunction and in different linear orders.

The prosodic boundary is phonetically realized through the location of the pause and final lengthening—the comparatively longer duration of the last pre-pausal syllable. Acoustic evidence for final lengthening has been reported in many languages, such as English (Cole et al., 2010), Dutch (Cho & McQueen, 2005) and Mandarin (Cao, 2004). Many studies have also reported longer pause durations at the end of a discourse unit than at the end of a prosodic phrase (Smith, 2004; L. Zhang et al., 2014, 2016). Final lengthening refers to an increase in the segmental duration at the right edge of different types of prosodic domains above the word level (Jun, 2018). It is an important cue for prosodic boundaries in speech, along with pauses and other cues, such as pitch reset (Petrone et al., 2017).

Other acoustic correlates of prominence include F0 (Katz & Selkirk, 2012; Wells, 2018; Xu, 1999; Xu & Xu, 2005) and an increased duration and intensity (Y. Mo et al., 2010). In research on Mandarin Chinese, scholars have obtained different rankings for acoustic correlates of stress. Some scholars believe that F0, duration, and intensity functions are unbalanced in the intonation of naturally spoken Chinese. Experiments have proven that most of the stress information in sentences is acoustically carried out by F0 and duration (Jia, 2012). Some scholars believe that, in Mandarin Chinese, the duration contributes the most to the perception of prosodic word stress, followed by pitch and spectral tilt (Zhong et al., 2001). Although scholars have different rankings for the importance of the acoustic correlates of stress, they recognize that pitch and duration are the two most important parameters.

Causality-related prosodic studies have focused on their role in distinguishing between different types of causal relations in English (Hu et al., 2022, 2023). Current research on causal prosody has primarily examined the objectivity and subjectivity of causal relationships. Speakers tend to use prosodic cues to reflect the objectivity and subjectivity of causality. When expressing subjective reasons, speakers seem to make more of an effort to convey information, which is specifically manifested in the fact that subjective causal clauses are more likely to reset pitch, have a more extensive pitch range, and set pauses to separate subjective reasons from result parts, forming two parallel intonation units.

With this understanding of explicit prosody, we now turn to the interplay between implicit an explicit prosody, which forms the basis of our experimental design.

### 2.4. Explicit Versus Implicit Prosody

Prosody can be generally divided into implicit and explicit prosody (Frazier & Gibson, 2015; Jun, 2010). Explicit prosody refers to actual, physically produced prosodic features in spoken language, such as intonation, stress, and pausing as just mentioned, while implicit prosody refers to the mental projection of prosodic patterns during silent reading or comprehension. As posited by the Implicit Prosody Hypothesis (IPH) (Fodor, 1998, 2002), the prosodic pattern mentally projected during silent reading is presumed to mirror the explicit prosody articulated in a comparable spoken context. This supposition is predicated on the notion that prosodic features generated internally during silent reading are analogous to those overtly produced in speech, suggesting that the study of explicit prosody can inform our understanding of implicit prosody. This hypothesis has been supported by many studies, including the role of stress information at the lexical level (Ashby & Clifton, 2005; Breen et al., 2019; Breen & Clifton, 2011, 2013) in lexical processing, as well as the impact of sentence-level prosodic manifestations such as prosodic boundaries (Bishop, 2021; Hwang & Steinhauer, 2011; Jun & Bishop, 2015; Swets et al., 2007) and intonation (Abramson, 2007) on sentence processing.

Meanwhile, an increasing number of psycholinguistic and neurophysiological experiments have suggested that the relationship between implicit prosody and explicit prosody is complex (Hirotani et al., 2006; Jun & Bishop, 2015; Schremm et al., 2015; Swets et al., 2007). As Jun (2010) points out in her discourse on the topic, a substantial amount of data support the idea that reading out loud is more closely linked to phonological processing, whereas reading silently is more closely connected to semantic processing. In laboratory settings, where speakers are asked to read the materials out loud, speakers tend to focus more on the fluency and rhythm of their speech rather than the encoding of syntactic and semantic elements, especially when the reading materials are lengthy or intricate (Rasinski et al., 2009). This tendency can be further intensified when the sentences are read in isolation, because the processing of meaning is less efficient without the support of a broader semantic or pragmatic context (Turnbull et al., 2017). In this scenario, can the pragmatic focus effect on the cause clause led by *yinwei* be realized through implicit prosody?

### 2.5. Gaps and Research Questions

The literature reveals that connectives, clause order, and prosody shape causal comprehension, yet no study integrated these to explore how *yinwei* signals pragmatic focus across spoken and silent modalities in a paratactic language. This study addresses this gap with two questions:

RQ1: How is the pragmatic focus on the *yinwei*-led cause clause acoustically realized in spoken Chinese?

RQ2: How do explicit and implicit prosodic patterns of primary stress and acoustic cues (pitch, duration) in *yinwei* sentences align, and do they support the Implicit Prosody Hypothesis?

Given these gaps—particularly the lack of prosodic analysis of *yinwei* in Mandarin Chinese—we conducted two experiments to explore its effects in both silent and spoken contexts.

## 3. Materials and Methods

### 3.1. Materials

To answer the research questions, we designed materials for two reading tasks: a silent reading/implicit focus identification task in a questionnaire (to access implicit focus position) and a reading aloud task (to assess explicit focus position). Table 1 shows a simple sentence structure used as the parental sentence (with None being the sentence with no conjunction to indicate a logical relation): “She/He + will + Verb, so/because + he/she + will + Verb”. We used third-person singular pronouns as the subjects in each clause because they share the same Chinese pronunciation but refer to different genders. Four sentences with verbs in four tones were designed for each type. To ensure a balanced distribution of subjects across the four tones, in sentences with the first and second tones, “he” was the subject of the cause clause; in sentences with the third and fourth tones, “she” was the subject of the cause clause. Only one conjunction, either *suoyi* (so) or *yinwei* (because), was used before the second clause to indicate the linear order of cause–effect (CE) or effect–cause (EC), which is consistent with the pattern found in the dialogue corpus.

### 3.2. Participants

Thirty native speakers of Mandarin Chinese (15 female, 15 male; age M = 20.2 years) were recruited from undergraduates in the university. All reported normal or corrected-to-normal vision, no history of language, reading, speech, or hearing impairments, and no prior exposure to the specific sentences or tasks used in this study. Participants provided informed consent in accordance with the institution’s ethics guidelines and received ¥50 after the experiment.

### 3.3. Procedure

This study comprised two sequential phases—an implicit prosody (silent-reading) experiment followed by a production (read-aloud) experiment—each designed for full reproducibility. 

For the silent reading/implicit prosody experiment, the first task was to choose the most suitable conjunction for complexes in None condition, with the three other conjunctions listed indicating other logical relations, such as progressive (*budan…*, *erqie…*, not only… but also…), conjunction (*bingqie…*, and…), and condition (*ruguo…*, *name…*, if…, then…). The participants could choose more than one option. They could also provide other logical relations, if necessary.

Their second task in silent reading was to select the most prominent syllable in each sentence. The materials included three target sentences and seven filler sentences in other logical relations. The participants were asked to choose the syllable they perceived as most prominent (i.e., carrying the focus). Wenjuanxing (https://www.wjx.cn), an online questionnaire platform, was used to collect data during silent reading sessions.

In the production experiment, all participants read aloud twelve target sentences (three sentence structures × four tones) and 96 filler sentences with other semantic relations all matched for length and syntactic complexity. Recordings took place in a sound-attenuated booth using MyRecord software (2.0) and AKG C-1000s and AKG-4000B microphones (manufactured by AKG Acoustics, in Vienna, Austria) in stereo mode (41 kHz sampling rage, 16-bit resolution). As the sentences were syntactically simple, we anticipated that participants might read them monotonously, without fully constructing their meanings. To counteract this, a lab assistant was stationed outside the recording booth and visible through a window. Participants were instructed to read each sentence clearly and naturally so that the assistant could grasp their intended meaning. By creating this “audience” context, we encouraged speakers to actively build and convey the semantic relationship between clauses rather than merely reciting words.

### 3.4. Data Analysis

#### 3.4.1. Silent Reading Task

We exported the results directly from the questionnaire platform. A clause with a prominent syllable was considered a prominent clause. In task one, over 53% of the participants considered causal relations to be the most suitable and natural for the non-conjunction condition. Meanwhile, 39% chose a progressive relation, 29% chose a conjunction relation, and 16% chose a conditional relation. The no-conjunction condition served as a baseline to assess default stress patterns, though its causal interpretation was ambiguous (53% participant agreement), limiting direct comparisons with CE and EC conditions.

#### 3.4.2. Production Task

We obtained 120 speech samples (30 participants × 4 tones) for each target condition for further statistical analysis. The sound files were labeled and analyzed using the Praat speech analysis software (version 6.1), which included the following steps.

First, we used an automatic labeling script in Praat to segment and label the boundaries of each syllable and pauses in the sentences. The TextGrids were then manually checked. Sentence stress in the target was manually labeled, with the most prominent stress was chosen as the primary stress. A clause carrying the primary stress was regarded as the more salient clause in the complex sentence. The pitch file (PitchTier) was manually modified to remove the pitch values corresponding to consonants and incorrect creaky voice segments to ensure that Praat exported accurate pitch data. Praat scripts were used to automatically extract the labeled durations and pitch values for each target sentence, with ten equidistant points of F0 extracted for each syllable.

The individual causal sentence duration and pitch data for each participant were normalized using Z-scores, and the normalized data were further analyzed using a Linear Mixed-Effect Model (LMM). Z-score normalization was used. The formula used is as follows:

Z-score = (Target Value − Mean Value)/(Standard Deviation)

With this experimental framework, we aimed to uncover *yinwei*’s prosodic influence across modalities. The following section presents the outcomes of these tasks.

## 4. Results

### 4.1. Prosodic Boundary in Explicit Prosody

We first analyzed the prosodic boundaries in explicit prosody, focusing on the prosodic boundary as an indicator of the clause structure. The parameters related to the prosodic boundary include the duration of the pause between the two clauses, the extension of the final syllable (final lengthening), and changes in pitch, including pitch declination and pitch reset. In the CE and EC conditions, the second clause was initiated by the conjunctions “so” and “because”, respectively, and they did not match in tone; thus, we cannot rigorously compare the pitch declination or reset at the beginning of the second clause. Therefore, in terms of prosodic linking patterns, we examined two main parameters: the pause between the two clauses and the lengthening of the final syllable in the first clause.

The variable pauses between clauses refers to the pause duration between the first and second clauses. According to the corpus design, there should be 120 recordings in each condition (four tones × 30 speakers), resulting in a total of 360 pause durations. However, in the actual reading, not every speaker paused between the clauses in the different target sentences. In the CE condition, there were 13 target sentences without a pause, and in the EC condition, there were nine target sentences without a pause. Thus, we obtained 338 pause durations (120 for the no-conjunction condition, 107 for the CE condition, and 111 for the EC condition).

Table 2 presents the mean and standard deviation of the duration of the pauses between the clauses and the final lengthening for the three conditions. We applied the Linear Mixed-Effects Model (LMM) to analyze the differences with the random effects of speaker ID and sentence ID.

The results of the LMM analysis indicate significant differences in the duration of the pauses between the clauses in the three conditions (None vs. CE: *β* = −122.9, SE = 12.7, t = −9.649, *p* < 0.001; None vs. EC: *β* = −36.9, SE = 14.7, t = −2.513, *p* = 0.031; CE vs. EC: *β* = −83.7, SE = 9.72, t = −8.608, *p* < 0.001). In addition to longer pauses in the EC condition, we observed no significant lengthening in the CE condition, suggesting a context-dependent effect.

No significant differences were found in the final pre-pausal syllable lengthening at the end of the first clause in the three conditions (None vs. CE: *β* = 8.66, SE = 4.43, t = 1.952, *p* = 0.14; None vs. EC: *β* = 3.83, SE = 5.71, t = 0.67, *p* = 0.535; CE vs. EC: *β* = 4.99, SE = 4.87, t = 1.026, *p* = 0.319).

### 4.2. Primary Stress Assignment

Having examined the boundary cues, we then explored the primary stress assignment as a marker of the pragmatic focus in both implicit and explicit prosody. In the silent reading, the participants’ task was to identify the position of the primary stress, where they needed to select the word they perceived as the most emphasized. Correspondingly, even though the pause duration between the two clauses was relatively long in the production experiment (with a mean range of 188–300 ms), which did not rule out the possibility that some speakers might consider them two prosodic phrases, we still only chose the syllable that sounded the most prominent as the primary stress of the target sentence.

**Figure 1:**
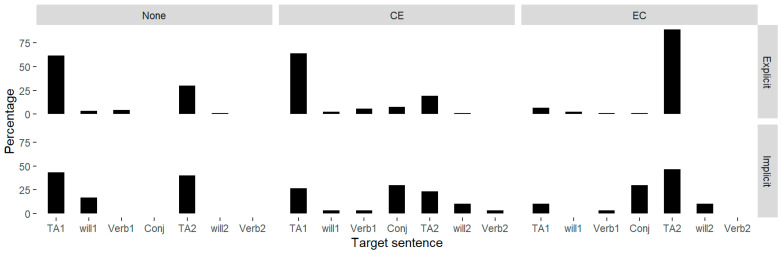
The percentage of each character being assigned a primary stress in implicit and explicit reading.

The bar chart in Figure 1 shows the probabilities of different characteristics carrying the primary stress under the three conditions. In the explicit reading, it was shown that most of the primary stresses were on the subjects of the two minor clauses, “TA1/TA2”, which was also the contrastive focus in the complex sentence. Unlike the patterns in explicit reading, the focus assignment showed a varied distribution in the implicit reading. In the CE and EC conditions, the conjunctions *suoyi* (so) and *yinwei* (because) showed a much higher percentage of prominent stress in the silent reading than in the actual prosodic realization.

**Figure 2:**
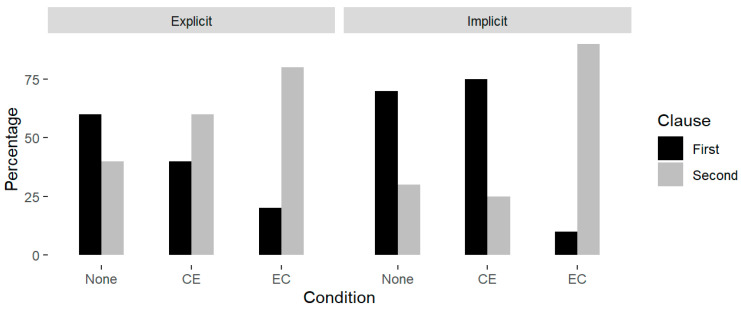
Percentage of the prominent syllable in explicit and implicit prosody across conditions.

When assigning the primary stress, we considered the clause carrying the primary stress as the emphasized clause, and we conducted a statistical analysis of the primary stress assignment pattern. As shown in Figure 2, we calculated the percentage of the two clauses carrying the primary stress in the three conditions of implicit and explicit prosody. We distinguished the order of the clauses as first or second clauses with different colors.

#### 4.2.1. Which Clause Is More Emphasized: The First or the Second?

The first question addressed the probability of the primary stress falling on each clause. As each sentence has only one primary stress and one emphasized word, the clause is more likely to bear this emphasis. To answer this question, we examined the differences in the probability of each clause receiving the primary stress across each experiment and condition. We chose the chi-square test because each of the 30 participants made only one selection, and from a data-volume perspective, the chi-square test can better describe the differences in the data. The results of the chi-square test are as follows.

In the questionnaire selection, in the no-conjunction and CE conditions, there was no significant difference in the probability of the first and second clauses being emphasized (χ^2^_None_(1) = 3.240, *p* = 0.072; χ^2^_CE_(1) = 3.240, *p* = 0.072). In the EC condition, the second clause (cause clause) had a higher probability of being emphasized (χ^2^(1) = 40.960, *p* < 0.001).

In the acoustic production experiment, there were significant differences in the probability of the first and second clauses receiving the primary stress under each condition ((χ^2^_None_(1) = 14.44, *p* < 0.001; χ^2^_CE_(1) = 21.160, *p* < 0.001; χ^2^_EC_(1) = 64, *p* < 0.001). However, these patterns were not the same. In the no-conjunction and CE conditions, the first clause had a higher probability of receiving the primary stress. In contrast, the EC condition showed a different pattern: the second clause had a significantly higher likelihood of being assigned the primary stress than the first clause.

#### 4.2.2. Which Is More Emphasized: The Cause Clause or the Effect Clause?

We used the primary stress assignment pattern in the no-conjunction condition in the questionnaire selection and acoustic production as a reference, and we compared the primary stress assignment patterns in the CE and EC conditions with the no-conjunction condition.

First, we compared the differences in the primary stress assignment patterns of CE and EC in the questionnaire selection and production experiments. We found that, due to the different linear orders, there were significant differences in the primary stress assignment patterns (χ^2^_implicit_(1) = 12.718, *p* < 0.001; χ^2^_explicit_(1) = 81.742, *p* < 0.001), and both showed a higher proportion of the cause clauses receiving the primary stress. While the EC condition showed a consistent stress on the cause clause, the CE condition revealed a split, with implicit prosody favoring the effect clause.

As mentioned earlier, since we cannot be certain that a causal relationship is established for the no-conjunction condition, we considered it as a baseline in the statistics to compare the CE and EC conditions. In response to the previous question, we found that in the no-conjunction condition, the first clause had a higher probability of receiving the primary stress, showing a front-heavy pattern, which we call the “front-focused pattern”. When compared with the EC condition, if we predict that the cause clause in the second clause is more emphasized and has a higher probability of receiving the primary stress, showing a “back-focused pattern”, then the chi-square test will show a significant difference. In the CE condition, the cause clause was in the first clause, and if the CE condition also showed a heavier pattern in the first clause, we believe there may be two reasons: one is consistent with the “front-focused pattern” in the no-conjunction condition and the other is due to the emphasis on the cause clause.

The chi-square test proves that there is a significant difference in the primary stress assignment pattern between the EC and non-conjunction conditions (χ^2^_implicit_ (1) = 35.498, *p* < 0.001; χ^2^_explicit_(1) = 35.498, *p* < 0.001), so we believe that in the EC condition, the pragmatic focus effect of the cause clause led by the conjunction “because” was reflected.

In the comparison between the CE condition and the no-conjunction condition, we found that in the questionnaire selection, there was a significant difference in the stress assignment patterns between the two conditions (χ^2^ (1) = 6.48, *p* = 0.011) with the opposite patterns; in the production experiment, there was no significant difference in the patterns (χ^2^ (1) = 6.48, *p* = 0.011), with the first clause having a higher probability of bearing the primary stress than the second clause.

#### 4.2.3. Is the Logic Focus Effect of the Causal Conjunction Consistent in Implicit and Explicit Prosody?

To answer this question, we compared the primary stress assignment patterns between the experiments in the CE and EC conditions.

In the EC condition, the primary stress assignment pattern showed a very consistent performance in both experiments (χ^2^ (1) = 2.658, *p* = 0.103), demonstrating that the cause clause (the second clause) had a higher probability of receiving the primary stress.

In the CE condition, there was a noticeable difference in the stress patterns between implicit and explicit prosody (χ^2^ (1) = 20.889, *p* < 0.001), with more participants in the questionnaire considering the second clause to be more emphasized (but not statistically significant). In contrast, the actual acoustic production stress distribution pattern was reversed, with the first clause having a significantly higher probability of receiving the primary stress than the second.

### 4.3. Prosodic Patterns of Focus in Causal Complex Sentences

To further clarify these patterns, we next investigated pitch and duration as acoustic correlates of focus in the production task. We examined the prosodic realization of focus in complex sentences expressing causal relationships. We used the pitch and duration of the two subjects as the parameters for analyzing the prosodic patterns of focus and used the LMM for analysis. The fixed effects were conjunctions (with or without), linear order, and clause position (first or second clause), whereas the random effects were speaker ID and sentence ID. There were 720 tokens in the three conditions, with 120 audio files per condition (4 tones × 30 speakers).

#### 4.3.1. Pitch Analysis

**Figure 3:**
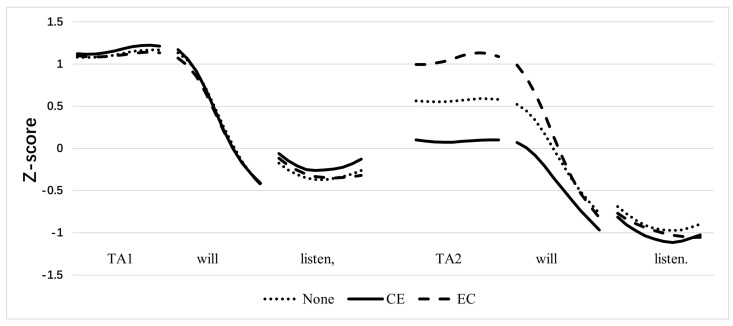
Normalized pitch contours of the target sentences.

We first examined the pitch contour with a verb in tone 1 under different conditions, as shown in Figure 3. The pitch contours of the *suoyi* and *yinwei* conjunctions in CE and EC were removed. The vertical axis represents the average Z-score-normalized pitch of each speaker.

From the pitch contours in the three conditions shown in Figure 3, we can observe that in None and EC, the pitch of TA2 exhibited varying degrees of declination. However, in EC, the pitch of TA2 was not significantly different from that of TA1. We also conducted a further statistical analysis on the position of the primary stress in the acoustic output and the probability of each position receiving the primary stress.

Since the third-person singular pronoun in Chinese is a level tone, the mean value was regarded as the pitch of each token from the ten points extracted from each character. Table 3 presents the mean F0 and standard deviations of TA1 and TA2 under these conditions. Table 4 presents the results of the LMM.

First, we conducted an LMM analysis of the pitches of TA1 and TA2 under each condition. The results show significant differences in the sentences without conjunctions and in the CE order, with a noticeable decline in pitch for TA2 (No conjunction: *β* = 0.55, SE = 0.058, t = 9.397, *p* < 0.001; CE: *β* = 1.08, SE = 0.058, t = 18.411, *p* < 0.001). However, in the EC order, there was no significant difference in pitch between the cause clauses TA2 and TA1 (EC: *β* = 0.044, SE = 0.063, t = 0.71, *p* = 0.478).

Subsequently, TA1 and TA2 were compared under these conditions. The LMM analysis revealed no significant differences in the pitch of TA1 across the three conditions (No conjunction vs. CE: *β* = −0.04, SE = 0.053, t = −0.898 *p* = 0.37; No conjunction vs. EC: *β* = 0.01, SE = 0.054, t = 0.187, *p* = 0.852; CE vs. EC: *β* = 0.058, SE = 0.054, t = 1.08, *p* = 0.281). By contrast, the pitch of the latter character, TA2, showed significant differences. Pairwise comparisons indicated significant differences in pitch in each condition (No conjunction vs. CE: *β* = 0.48, SE = 0.062, t = 7.71 *p* < 0.001; No conjunction vs. EC: β = −0.49, SE = 0.067, t = −7.4, *p* < 0.001; CE vs. EC: *β* = −0.97, SE = 0.066, t = −14.715, *p* < 0.001), with the most pronounced declination in pitch for TA2 in the CE condition. In the EC condition, the pitch of TA2 increased significantly owing to the effect of pragmatic focus.

#### 4.3.2. Duration Analysis

Table 5 presents the average duration and standard deviation of TA1 and TA2 in isolated sentences. We also conducted an LMM statistical analysis of duration with three factors: conjunctions, word order, and clause position. The results are shown in Table 6.

From Table 6, we can observe significant interaction effects between the conditions and positions of the clauses on the duration.

First, we conducted an LMM analysis to examine the differences in duration between TA1 and TA2 under the same condition. The results show no significant difference in the duration between TA1 and TA2 in the sentences without conjunctions and in the CE order (No conjunction: β = 0.0008, SE = 0.005, t = 0.162, *p* = 0.872; CE: β = 0.0029, SE = 0.005, t = 0.608, *p* = 0.544). However, in the EC order, the duration of TA2 was significantly lengthened (EC: *β* = −0.024, SE = 0.004, t = −5.085, *p* < 0.001), indicating the dual effects of contrastive and causal pragmatic focus.

Next, we compared TA1 and TA2 across the conditions to explore the differences between the conditions. The LMM analysis revealed no significant differences in the duration of TA1 in the CE condition compared with the other two conditions (No conjunction vs. CE: *β* = 0.004, SE = 0.005, t = 0.927, *p* = 0.355; CE vs. EC: *β* = 0.006, SE = 0.005, t = 1.384, *p* = 0.168). However, the duration of TA1 in the EC condition was significantly shorter than in the no-conjunction condition (No conjunction vs. EC, *β* = 0.01, SE = 0.004, t = 2.324, *p* = 0.021). The latter character, TA2, showed significant differences. Pairwise comparisons indicated no significant differences in the duration of TA2 between the no-conjunction and CE conditions (*β* = 0.007, SE = 0.005, t = 1.395, *p* = 0.164); in contrast, TA2 in the EC order—that is, the cause clause’s TA2—was significantly longer than TA2 in the non-conjunction condition (*β* = −0.139, SE = 0.005, t = −2.754, *p* = 0.006) and in the CE order (*β* = −0.02, SE = 0.004, t = −4.269, *p* < 0.001).

These results highlight *yinwei*’s robust focus effect, particularly in the EC condition. In the next section, we interpret these findings in relation to the IPH and Mandarin’s syntax.

## 5. Discussion

This study investigated the prosodic realization of the pragmatic focus in Chinese causal complex sentences, focusing on the conjunction *yinwei* (because) and its interplay with implicit and explicit prosody. Our findings reveal that *yinwei* robustly marks the cause clause as pragmatically salient through distinct acoustic cues—longer pause durations, higher pitch, and extended syllable duration—particularly in the EC order. These results not only extend prior research on causal connectives (Canestrelli et al., 2016; T. J. M. Sanders & Noordman, 2000), but they also challenge the assumptions about prosodic alignment across reading modalities, offering new insights into Mandarin discourse processing.

### 5.1. Prosodic Realization of Pragmatic Focus

Addressing our first research question, we found that *yinwei* enhances the cause clause’s salience through distinct prosodic cues.

In explicit prosody, the EC condition displayed significantly longer pause durations before the cause clause (M = 271.85 ms) compared to the cause–effect (CE) condition (M = 188.05 ms, *p* < 0.001), highlighting the pauses as key indicators of pragmatic focus (Zhao, 2012). These findings align closely with our previous reading-production experiment using 72 monologues containing causal relations (42 words, three clauses, read out loud by 30 university students) (L. Zhang, 2022). In that study, the internal pause durations in the CE condition were significantly shorter than in the EC condition, reflecting a tighter segmentation when the discourse order matched real-world causality. Pause placement is known to correlate strongly with syntactic structure (Selkirk, 2011) and with semantic cohesion, as predicted by models of discourse processing such as Grosz and Sidner’s Attention Model (1986), Asher and Vieu’s Segmented Discourse Representation Theory (Asher & Vieu, 2005), and Rhetorical Structure Theory (Mann & Thompson, 1988; L. Zhang et al., 2014, 2016).

Interestingly, the final-syllable lengthening showed no notable variation across the conditions (*p* > 0.14), suggesting that the pause duration serves as a more reliable boundary cue in Mandarin—a pattern consistent with cross-linguistic observations (Fon et al., 2011). This reliance on pauses may reflect Mandarin’s paratactic nature, where explicit subordinating markers are scarce, and prosody assumes a heightened role in delineating the clause boundaries and signaling the discourse structure. The CE condition’s divergence may reflect *suoyi*’s role as a forward-looking connective, prompting implicit readers to focus on the effect clause for narrative resolution (Canestrelli et al., 2016), while explicit prosody emphasizes the cause clause due to phonological demands.

The pitch and duration analyses further illuminated the focus realization. In the EC condition, the subject of the cause clause (TA2) exhibited a higher pitch (M = 1.066 Z-score) and a longer duration (M = 0.226 Z-score) compared to the CE condition (pitch: M = 0.088, *p* < 0.001; duration: M = 0.206, *p* < 0.001). This suggests that *yinwei* amplifies the prosodic prominence of the cause clause, aligning with its pragmatic importance (L. Zhang, 2022). In contrast, the CE condition showed a marked pitch declination in the effect clause (TA2), potentially due to the conjunction *suoyi* (so) initiating the second clause, which may inhibit pitch reset. These patterns clarify the ongoing debates about clause focus in Chinese causal constructions (Huang & Liao, 2007; W. Li, 2018), demonstrating that linear order and conjunction type are pivotal in shaping the focus assignment.

The prominence of prosodic cues in the EC order underscores Mandarin’s paratactic structure, where clauses are frequently juxtaposed without overt connectives (Bookheimer et al., 1995). Unlike hypotactic languages that rely on subordinating conjunctions (e.g., English “because”), Mandarin often depends on the context and prosody to convey clause relationships (N. Li & Thompson, 1981). The strategic use of *yinwei* in the EC order exemplifies how an explicit marker can guide the prosodic focus, compensating for the language’s inherent implicitness. For instance, in a sentence such as *ta1 lei4 le5*, *yinwei gong1 zuo4 dao4 hen3 wan3* (She was tired because she worked late), the pause before *yinwei* and the pitch rise after *yinwei* signal the explanatory focus, a subtlety that might be lost in paratactic juxtaposition alone.

### 5.2. Implicit vs. Explicit Prosody

The Implicit Prosody Hypothesis posits that prosodic contours in silent reading mirror those in spoken production. Our findings provide nuanced support for this hypothesis. In the EC condition, both implicit and explicit prosody consistently emphasized the cause clause (χ^2^ = 2.658, *p* = 0.103), indicating that *yinwei*’s focus effect persists across modalities.

Whereas the EC condition aligns with the IPH, the CE condition’s divergence suggests modality-specific influences on prosodic projection. In the CE condition, implicit prosody favored the effect clause (second clause), while explicit prosody highlighted the cause clause (first clause, χ^2^ = 20.889, *p* < 0.001). This divergence may stem from differing cognitive demands: silent reading prioritizes semantic coherence (Bookheimer et al., 1995), while reading aloud emphasizes phonological clarity (Rasinski et al., 2009). The elevated stress on *suoyi* in implicit reading (28% vs. 8% in explicit reading) suggests that silent processing may heighten conjunction salience, possibly due to task-specific repetition.

These results refine the IPH by pinpointing contextual variables—such as conjunction type, linear order, and modality—that influence prosodic alignment. They also contribute to cross-linguistic evidence (Ford, 1994) by showcasing how Mandarin’s tonal system and flexible syntax introduce distinct prosodic dynamics. The discrepancy in the CE order challenges the universality of implicit prosody (Breen et al., 2019) and hints at the impact of Mandarin’s paratactic structure. In paratactic languages, where explicit subordination is less common, silent readers may lean more heavily on semantic cues, projecting prosodic patterns that diverge from spoken norms. For example, in the CE-order complex sentence *Yinwei ta1 e4 le5*, *ta1 chi1 le5* (Because he was hungry, he ate), implicit prosody might emphasize the effect “*ta1 chi1 le5*” (he ate) as the logical conclusion, whereas reading aloud foregrounds the cause “*yinwei ta1 e4 le5*” (because he was hungry) via prosodic cues.

### 5.3. Theoretical and Practical Implications

Theoretically, this study highlights prosody’s pivotal role in Mandarin, a language shaped by its tonal system, paratactic structure, and sparse use of explicit markers. In contrast to hypotactic languages that employ subordinating conjunctions to clarify clause relationships (e.g., English, German), Mandarin’s parataxis—juxtaposing clauses without connectives—relies on word order, context, and prosody to convey meaning (N. Li & Thompson, 1981). Our evidence that *yinwei* enhances cause clause salience via prosodic cues illustrates how Mandarin compensates for its minimal marking. This challenges universalist discourse models (e.g., T. J. M. Sanders & Noordman, 2000), which often presuppose explicit connectives, urging a rethinking of coherence mechanisms across linguistic typologies.

Mandarin’s paratactic nature also fuels scholarly debates about linear order and discourse processing (Biq, 1995; H. Li, 2012). The preference for the EC order with *yinwei* may reflect a pragmatic choice to foreground explanatory content in a language where explicit subordination is rare. This prompts a broader question: Does parataxis inherently elevate prosody’s role in distinguishing clause types? Chao (1979) attributes Mandarin’s parataxis to a cultural penchant for implicitness, while Tsao (1990) views it as a structural trait necessitating prosodic support. We argue that prosody’s prominence in focus marking may be a universal feature of paratactic languages, though Mandarin’s tonal complexity could amplify this effect. Comparative studies with other paratactic (e.g., Japanese, Thai) and hypotactic (e.g., Russian, Arabic) systems could test this hypothesis, potentially refining global theories of prosody and pragmatics.

These insights extend beyond theory, offering practical applications for language processing and education. In NLP, Mandarin speech synthesis must prioritize prosodic modeling—factoring in pause duration, pitch variation, and syllable length—to capture the language’s paratactic subtleties. Our data suggest that algorithms should integrate prosodic and syntactic features to improve text-to-speech naturalness, especially for causal constructions. In language pedagogy, teaching Mandarin to non-native speakers, particularly those from hypotactic backgrounds, requires an emphasis on prosodic cues and paratactic patterns. Exercises contrasting *yinwei* in the EC order (*Ta1 mei2 lai2*, *yinwei bing4 le5* (He didn’t come because he was sick) with paratactic equivalents (*Ta1 bing4 le5*, *ta1 mei2 lai2* (He was sick, he didn’t come) could enhance the learners’ grasp of focus and coherence.

### 5.4. Limitations and Challenges

Several limitations temper our conclusions. First, the use of controlled sentences with a single conjunction (*yinwei* or *suoyi*) limits the generalizability to naturalistic discourse, where varied connectives and complex structures are common. This design choice, while necessary for experimental control, may have amplified prosodic effects relative to spontaneous speech. Second, the participant sample (30 university students, mean age 20.2 years) was small and homogeneous, potentially overlooking age, dialect, or socioeconomic variations in prosodic realization. Third, the reading-aloud task’s instruction to ensure clarity for a lab assistant may have exaggerated prosodic features, as the participants prioritized intelligibility over natural intonation. Finally, the silent reading task allowed multiple readings, which may have inflated the semantic focus on conjunctions, particularly *suoyi*.

These limitations reflect the practical constraints but also highlight the methodological challenges. For instance, balancing the experimental control with ecological validity remains a persistent issue in psycholinguistic research. We mitigated this by using simple, high-frequency sentences, but future studies must grapple with capturing prosody in less controlled settings, such as conversational situations, which poses logistical and analytical hurdles.

### 5.5. Future Directions

To address these limitations, future research should explore prosodic focus in naturalistic Mandarin discourse, incorporating diverse connectives (e.g., *youyu*, *jiran*) and complex sentence structures. Cross-linguistic studies comparing Mandarin with non-tonal languages (e.g., English, Spanish) could clarify whether *yinwei*’s focus effect is language-specific or universal, testing the IPH’s generalizability. Neurophysiological approaches could probe the cognitive mechanisms underlying implicit–explicit prosodic differences, offering insights into the neural basis of focus processing. Additionally, applied research could develop prosodic training programs for Mandarin learners, leveraging our findings to improve spoken fluency.

We also see value in exploring cultural influences on prosodic focus. Mandarin speakers may prioritize explanatory content due to cultural norms around reasoning and argumentation, a hypothesis that could be tested through comparative studies with other East Asian languages (e.g., Japanese, Korean). Such work could bridge linguistics with cultural psychology, fostering interdisciplinary dialogue.

## 6. Conclusions

In summary, our findings demonstrate that *yinwei* shapes prosodic patterns in Mandarin, refining our understanding of the IPH in paratactic languages. This study demonstrated that in Mandarin Chinese—a tonal, paratactic language with minimal explicit grammatical markers—the conjunction *yinwei* in effect–cause order robustly signals the cause clause’s pragmatic salience through prosodic features: extended pause durations, heightened pitch, and prolonged syllable lengths. These findings elucidate how Mandarin harnesses prosody to offset its limited use of explicit connectives, offering partial validation for the Implicit Prosody Hypothesis while revealing its contextual constraints. By refining models of discourse coherence and prosodic focus, this research underscores the importance of language-specific analyses in psycholinguistics, particularly for paratactic systems where prosody compensates for syntactic implicitness.

The paratactic structure of Mandarin amplifies prosody’s role, challenging universalist assumptions about discourse processing and inviting broader cross-linguistic inquiry. These insights not only advance the theoretical understanding—highlighting the interplay of prosody, syntax, and pragmatics—but also inform the practical applications in NLP and language teaching, where capturing Mandarin’s nuances is paramount. Future investigations should extend this work to naturalistic settings, diverse populations, and comparative frameworks, further unraveling the intricate dynamics of prosody in paratactic languages and beyond.

## Figures and Tables

**Table 1:** Sample target sentences (verbs in tone 1).

Condition	Sentence	Conjunction	Linear Order
None	他要听，她要听。	None	
	ta1 yao4 ting1, ta1 yao4 ting1.		
	He will listen, she will listen.		
CE	他要听，所以她要听。	So	CE
	ta1 yao4 ting1, suo3 yi3 ta1 yao4 ting1.		
	He will listen, so she will listen.		
EC	他要听，因为她要听。	Because	EC
	ta1 yao4 ting1, yin1 wei4 ta1 yao4 ting1.		
	He will listen, because she will listen.		

**Table 2:** Duration of pause and last pre-pausal syllable (ms).

	Pause Duration (ms)	Last Pre-Pausal Syllable (ms)
	M (SD)	M (SD)
None	302.85 (133.12)	212.17(47.80)
CE	188.05 (100.59)	208.41 (51.84)
EC	271.85 (114.84)	203.53 (47.47)

**Table 3:** F0 (M and SD) in the Z-score of the two pronouns.

	TA1		TA2	
	Mean	SD	Mean	SD
None	1.121	0.417	0.570	0.489
CE	1.169	0.419	0.088	0.483
EC	1.111	0.423	1.066	0.553

**Table 4:** Statistical results of the LMM of F0.

	Estimated	Standard Error	df	t	Sig.
Interception	1.066036	0.042419	714	25.131	0.000
Conjunction	−0.496403	0.059989	714	−8.275	0.000
Linear order	−0.978075	0.059989	714	−16.304	0.000
Clause position	0.044852	0.059989	714	0.748	0.455
Conjunction × clause position	0.506531	0.084838	714	5.971	0.000
Linear order × clause position	1.036580	0.084838	714	12.218	0.000

**Table 5:** The two pronouns’ durations (M and SD) in the Z-score.

	TA1		TA2	
	Mean	SD	Mean	SD
None	0.213	0.039	0.212	0.039
CE	0.208	0.038	0.206	0.036
EC	0.202	0.034	0.226	0.039

**Table 6:** Statistical results of the LMM of duration.

	Estimated	Standard Error	df	t	Sig.
Interception	0.226322	0.003420	714	66.180	0.000
Conjunction	−0.013930	0.004836	714	−2.880	0.004
Linear order	−0.020619	0.004836	714	−4.263	0.000
Clause position	−0.024112	0.004836	714	−4.986	0.000
Conjunction × clause position	0.024929	0.006840	714	3.645	0.000
Linear order × clause position	0.027014	0.006840	714	3.950	0.000

## Data Availability

The data presented in this study are available upon request from the corresponding author.
